# Bisphosphonate and medication induced necrosis of jaws

**DOI:** 10.6026/973206300210078

**Published:** 2025-01-31

**Authors:** Altaf H Malik

**Affiliations:** 1 Department of oral and Maxillofacial Surgery Government Dental College Srinagar-190010, Jammu and Kashmir, India

**Keywords:** Bisphosphonates, osteonecrosis, medication-related osteonecrosis of the jaw

## Abstract

The bisphosphonates are prescribed drugs to prevent bone resorption particularly in patients having certain malignancies. However, there is a potential risk of osteonecrosis of jaws in patients receiving dental treatment. Therefore, it is of interest to evaluate the chances of osteonecrosis in patients referred for management of osteonecrosis in whom dental treatment was carried out by the referring dental professionals. Hence, 86 patients were studied, where 18 patients had developed osteonecrosis particularly after dental extraction of which 13 patients were having underlying malignancy. We found that medication induced necrosis of jaws is more in patients with underlying malignancy or other co-morbidities. Moreover, extraction is a trigger in such patients for developing the debilitating condition.

## Background:

Marx and Stern described BRONJ (bisphosphonate related osteonecrosis of jaws) in patients on bisphosphonates for prevention of bone resorption; however an increasing number of cases are getting reported and recognized presently [1]. Due to increasing size of population with improvement of lifespan and unfortunate increased incidence of malignancies and other chronic bone wasting disease, the use of bisphosphonates and other anti-resorbative drugs has increased to counter the bone loss. BRONJ is defined as a condition where in there is exposure of bone for 8 or more than 8 weeks in mandible or maxilla in a patient who has been or are currently on bisphosphonates and have not received any radiation therapy. Bisphosphonates are currently exploited for the treatment of metastatic bone disease, hypocalcaemia of pregnancy, Paget's disease and other osteoporotic conditions. Very recently the literature suggests their role in osteonecrosis of jaws particularly after any sort of surgical or dental intervention particularly extractions of teeth in jaws [2, 3, 4- 5]. The American association of oral and maxillofacial surgeons changed the term BRONG to MRONG meaning medication related osteonecrosis is of jaws in 2014 as other drugs like densumab a RANKL ligand inhibitor and other antiangiogenic drugs were also found to be incriminated in the osteonecrosis of jaws [6]. MRONG is most commonly found in mandible with incidence rate of 68% compared to maxilla where it is 28% and it has around 4% incidence together [7]. Robert Marx in his study on patients of intravenous bisphosphonates observed about 75 %of Brong was post trauma due to some invasive dental procedure and 25 % of times it was spontaneous development particularly in patients with compromised oral hygiene [7]. Therefore, it is of interest to evaluate the chances of osteonecrosis in patients referred for management of osteonecrosis in whom dental treatment was carried out by the referring dental professionals.

## Materials and Methods:

The study was conducted in the department of oral and maxillofacial surgery on the patients on antireosbative drugs for different underlying medical condition and was referred for management of oral conditions after receiving dental treatments for their decayed or diseased teeth from 2021 to 2024. The study was approved by the institutional ethical committee with no OMFS 2021/47 and only such patients who were on injectable therapy for more than a year were included and followed, some of them had already developed osteonecrosis of jaws due to negligent dental treatement like extraction or curettage by their earlier dentist (picks 1, 2, 3) and were referred for further treatments in our department. In this way we studied 74 patients who were on antireosbative therapy. The study excluded patients who had received radiation or steroid therapy. The MRONG was diagnosed by clinical, radiological and histopathological examination to rule out any malignancy or recurrence. The patients were treated according to the protocol of stage of disease and were put on Vit E 500mg twice a day, pentoxyphyline 400 mg twice a day after cardiac clearance and according to the stage. Debridement, PRF therapy or resection of diseased part was carried out wherever needed. In 7 patients a platelet rich fibrin membrane was used after surgery as dressing and wound cover. The data was entered into SPS software and Chi Square test was applied for compilation.

## Results:

Out of 86 patients studied 68 patients had not developed osteonecrosis after dental treatement even after extraction in mandible/maxilla but all such patients were receiving bisphosphonates for osteoporosis. About 13 patients who had developed osteonecrosis were having underlying malignancy like of breast, prostrate, multiple myeloma (Table 1). The other 5 patients were having MRONG were receving bisphosphonates for osteoporosis. Of the 18 patients with MRONG mandible was involved as many as in 15 cases. In our series a total of 2.09% cases were found to have osteonecrosis and most of them were due to negligence and lack of information or knowledge by the earlier dentist is or quacks.

## Discussion:

BRONG or MRONG is new age epidemic due to increase in malignancy and other bone wasting diseases and negligence with lack of proper knowledge in patients and health care providers is additional risk factor. Though MRONG can develop spontaneously however about 80% develop post dental treatment particularly extractions. . The literature reports an incidence of 2.9% of MRONJ after tooth extraction in cancer patients and 0.15% in patients being treated for osteoporosis [8]. The other studies instead show that the incidence can be 18.6% in relation to the dose and time of administration of bisphosphonates in cancer patients [9, 10]. A review of 114 cases of bisphosphonates associated osteonecrosis of jaws in Australia showed that 73% of the cases occurred after dental extractions. The frequency of osteonecrosis of jaws in bisphosphonate treated osteoporotic patients was 0.01%-0.04% and if dental extraction occurred 0.09%-0.34%. In patients on bisphonates for bone malignancies, the incidence was 0.33%-1.15% and after dental extractions 6.7%-9.1% [11]. A holistic and multidisciplinary team approach for evaluation and management of the conditions is recommended including a dentist, an oral-maxillofacial surgeon and an oncologist and information about its usage should be given to masses and patients should be encouraged to inform about these drugs to their dentists before receiving the treatement of their ailments. In early stages, surgical debridement and coverage has been successful [12]. Osteotomies or resection are recommended only for severe cases [13, 14, 15- 16], due to relatively high levels of morbidity and impaired quality of life for the patients prevention is a cornerstone to reduce the incidence of osteonecrosis of jaws and before starting bisphosphonate therapy, the patient should be referred for thorough dental evaluation to identify and treat any potential source of infection. Start of bisphonate therapy should be delayed by 4-6 weeks to allow appropriate bone healing [17]. In our study we carried resection for one case for mandible, in other 17 cases conservative measures like local debridement, curettage and PRF therapy was done for the relief of symptoms and personal hygiene instructions. In our study a greater propensity was seen for progression to osteonecrosis of jaws in patients with underlying malignancy with p value of < 0.01 (Table 1). Prevention and pre-emptive surgical or other treatments are crucial before the start of the therapy [18]. The incidence of bisphosphonate induced necrosis varies according to underlying medical condition and is more seen in underlying malignancies [19]. The treatment of bisphosphonate-related osteonecrosis of the jaw needs comprehensive and holistic approach but it is better to launch a campaign about its information in the general public and concerned professionals. The fellow medical colleagues should educate about dental clearance before start of therapy to eradicate potential trigger for osteonecrosis of jaws in the form decayed, diseased teeth or poor oral condition.

## Conclusion:

Patients on anti-resorptaive medication are at increased risk of osteonecrosis particularly among those who have underlying malignancy. Hence, proper patient awareness among health care professionals is needed to prevent medication induced osteonecrosis.

## Figures and Tables

**Table 1:** The patients developing MRONG after treatment antireosbative drugs with underlying conditions

**N=86**	**Mrong Present**	**Mrong Absent**
Malignancy patients	13	0 (p<0.01)
Other than malignancy	5	68 (p>0.1)
